# The perception of speech modulation cues in lexical tones is guided by early language-specific experience

**DOI:** 10.3389/fpsyg.2015.01290

**Published:** 2015-08-28

**Authors:** Laurianne Cabrera, Feng-Ming Tsao, Huei-Mei Liu, Lu-Yang Li, You-Hsin Hu, Christian Lorenzi, Josiane Bertoncini

**Affiliations:** ^1^Centre National de la Recherche Scientifique, Laboratoire de Psychologie de la Perception, Université Paris DescartesParis, France; ^2^Department of Psychology, National Taiwan UniversityTaipei, Taiwan; ^3^Department of Special Education, National Taiwan Normal UniversityTaipei, Taiwan; ^4^Centre National de la Recherche Scientifique, Laboratoire des Systèmes Perceptifs, Institut d’Etude de la Cognition, Ecole Normale SupérieureParis, France

**Keywords:** speech perception, amplitude and frequency modulation, infants, lexical tones

## Abstract

A number of studies showed that infants reorganize their perception of speech sounds according to their native language categories during their first year of life. Still, information is lacking about the contribution of basic auditory mechanisms to this process. This study aimed to evaluate when native language experience starts to noticeably affect the perceptual processing of basic acoustic cues [i.e., frequency-modulation (FM) and amplitude-modulation information] known to be crucial for speech perception in adults. The discrimination of a lexical-tone contrast (rising versus low) was assessed in 6- and 10-month-old infants learning either French or Mandarin using a visual habituation paradigm. The lexical tones were presented in two conditions designed to either keep intact or to severely degrade the FM and fine spectral cues needed to accurately perceive voice-pitch trajectory. A third condition was designed to assess the discrimination of the same voice-pitch trajectories using click trains containing only the FM cues related to the fundamental-frequency (F0) in French- and Mandarin-learning 10-month-old infants. Results showed that the younger infants of both language groups and the Mandarin-learning 10-month-olds discriminated the intact lexical-tone contrast while French-learning 10-month-olds failed. However, only the French 10-month-olds discriminated degraded lexical tones when FM, and thus voice-pitch cues were reduced. Moreover, Mandarin-learning 10-month-olds were found to discriminate the pitch trajectories as presented in click trains better than French infants. Altogether, these results reveal that the perceptual reorganization occurring during the first year of life for lexical tones is coupled with changes in the auditory ability to use speech modulation cues.

## Introduction

In the first months of life, infants are able to discriminate almost all phonetic contrasts, including non-native ones (Kuhl, 2004; Werker and Tees, 2005). However, this early quasi-universal perceptual ability turns into a more native language-specific ability later in the first year of life. This so-called “perceptual reorganization” was initially demonstrated by Werker and Tees (1984) who found that young English-learning infants were able to discriminate a consonant contrast in Hindi between 6 and 8 months of age, but were unable to maintain this ability later on (between 10 and 12 months) while Hindi-exposed infants did so. Several studies replicated these initial results with other consonant contrasts using either behavioral or electrophysiological methods (e.g., Tsushima et al., 1994; Best et al., 1995; Cheour et al., 1998; Rivera-Gaxiola et al., 2005). This period of perceptual reorganization is not only marked by a declining discrimination ability for non-native contrasts: infants also become more accurate in discriminating phonetic contrasts of their native language during the same period, around their first birthday (e.g., Kuhl et al., 2006; Tsao et al., 2006; Conboy et al., 2008). This perceptual reorganization has been observed for consonant and vowel perception, with language-specific re-organization taking place around 6 months of age for vowel categories (e.g., Kuhl et al., 1992; Polka and Werker, 1994) and around 10 months for consonants. Together, these results demonstrate that speech perception is shaped by language experience during the first year of life, with infants becoming more and more sensitive to their native speech contrasts and less sensitive to non-native ones.

Compared to the perception of phonetic segments like consonants and vowels, the developmental time course of lexical-tone perception has received attention only recently. This is surprising given that more than 70% of the world’s languages include a (more or less complex) tone system (Yip, 2002). In (complex) tonal languages such as Thai or Mandarin, variations in fundamental frequency (F0) – i.e., voice pitch – within a syllable distinguish word meanings (e.g., Liang, 1963). In a pioneering study, Mattock and Burnham (2006) explored the ability to discriminate non-native Thai lexical-tone patterns in English- and Chinese-learning (either Mandarin or Cantonese) infants. The lexical-tone patterns consisted of the syllable /ba/ carrying a so-called Thai “contour-level” contrast (i.e., a low F0 syllable with a flat trajectory versus a syllable with a rising F0 trajectory) and a Thai “contour–contour” contrast (i.e., a syllable with a rising F0 trajectory versus a syllable with a falling F0 trajectory). Only English infants showed a decline in lexical-tone discrimination, for both contrasts, between 6 and 9 months of age. Moreover, the contour-level contrast was overall better discriminated than the contour-contour one. In comparison, all infants showed constant perceptual sensitivity to pitch variations conveyed by non-linguistic signals (i.e., musical tones played on a violin) between 6 and 9 months. In a subsequent study, Mattock et al. (2008) tested the discrimination of the same Thai contour-level lexical-tone contrast in 4- 6- and 9-month-old infants learning non-tonal languages (i.e., English or French). Despite rhythm differences between English and French (i.e., stress-timed versus syllable timed, respectively; Ramus et al., 1999), Mattock et al. (2008) found that both groups of non-native listeners behaved similarly regarding the discrimination of lexical tones. At 4 and 6 months of age, English and French-learning infants discriminated the contour-level lexical-tone contrast while at 9 months both language groups failed. Thus, for languages such as English or French where pitch variations at the syllable level have little relevance, infants become less sensitive to this variation between 6 and 9 months. Recently, Yeung et al. (2013) suggested that language-specific preferences for lexical tones may be found earlier, that is at 4 months, when infants learning two tonal languages (Mandarin or Cantonese) are compared. The developmental trajectory of lexical-tone perception remains to be explored and recent studies (see Liu and Kager, 2014) observed a perceptual rebound in lexical-tone discrimination by non-native listeners in the 2nd year of life. Altogether, these studies indicate that the perceptual reorganization for lexical-tone perception depends on early experience with a language having lexical tones. Before their first birthday, infants learn to perceive pitch variations at the syllable level as a reliable phonological cue when their native language is tonal.

Several factors may influence speech reorganization. Not only language experience, and thus, repeated exposure to the native phonetic categories and language regularities, but also cognitive (i.e., attentional processes related to executive and inhibitory control) and social skills (i.e., social interactions) have been shown to influence the development of phonetic discrimination (e.g., Saffran et al., 1996; Saffran, 2002; Kuhl et al., 2003; Conboy et al., 2008; see Kuhl, 2004, 2009 for a review). The development of phonetic discrimination can be also described as being driven by the intrinsic properties of the speech signal and by sensory constraints imposed by the human auditory system (Aslin and Pisoni, 1980; Nittrouer, 2002). Indeed, the development of discrimination abilities for infants has been shown to differ across speech contrasts (e.g., Pisoni, 1977; Aslin et al., 1981; Polka et al., 2001; Mattock and Burnham, 2006; Narayan et al., 2010). Furthermore, changes in the perceptual weight of specific acoustic cues in speech signals have been observed during development. For instance, between 4 and 8 months French-learning infants become more and more sensitive to the characteristic voice-onset-time (VOT) boundaries of their native language (e.g., Hoonhorst et al., 2009). An influence of language experience on the weight of suprasegmental cues signaling clausal boundaries (such as pause, pitch, and vowel duration, cf. Seidl, 2007; Seidl and Cristià, 2008) has also been observed between 4 and 6 months. Moreover, infants become sensitive to the spectral structure of their native language between 6 and 9 months of age. Beach and Kitamura (2011) tested the discrimination ability of English infants with altered speech sounds assigning prominence to either high-frequency or low-frequency information. While 6-month-olds discriminate the native speech sounds in all conditions, 9-month-olds discriminate only the unaltered versions. These studies suggest that with exposure to a given language, infants come to be more dependent on native language-specific acoustic features (and to specific configurations of acoustic cues) signaling both segmental and suprasegmental contrasts. This implies that the perceptual reorganization for speech may involve developmental changes of relatively basic auditory processes such as the processing of spectro-temporal cues of speech signal.

However, to our knowledge, no study investigated the *early perceptual re-weighting of these spectro-temporal cues* by comparing discriminative responses in infants from different language backgrounds. In adults, psychoacoustic studies repeatedly found that listeners differ in the nature (and probably in the weight) of the spectro-temporal cues they rely on according to the speech sounds they listen to and according to their native language. Over the last decades, an original psychoacoustic paradigm was developed to explore the auditory processing of speech acoustic properties, based on the notion that speech information is mainly conveyed by the temporal modulations at the output of cochlear filters (e.g., Steeneken and Houtgast, 1980). To test this notion, “vocoders,” which are speech analysis and synthesis systems, were used to manipulate the *modulation components* of speech in a given number of frequency bands (Shamma and Lorenzi, 2013). Here, each frequency band is viewed as a sinusoidal carrier with superimposed amplitude modulation (AM or acoustic “temporal envelope” cues corresponding to the slow variations in amplitude over time) and frequency modulation (FM or acoustic “temporal fine structure” corresponding to the oscillations in instantaneous frequency close to the center frequency of the band; e.g., Drullman, 1995; Shannon et al., 1995; Smith et al., 2002; Zeng et al., 2005; Sheft et al., 2008). The AM cues convey information about speech rhythm whereas FM cues convey (F0-related) pitch information (e.g., Rosen, 1992; Smith et al., 2002). Thus, when identifying syllables, words and sentences, English- and French-speaking adults rely mainly on the AM cues (e.g., Shannon et al., 1995; Smith et al., 2002). Not surprisingly, a different pattern of results was found for listeners identifying lexical tones in which pitch information conveys lexical meaning (e.g., Fu et al., 1998; Xu et al., 2002; Kong and Zeng, 2006). For adults using a tonal language, Xu and Pfingst (2008) showed that the identification of lexical tones is mainly based on FM cues. Furthermore, Cabrera et al. (2014) showed that Mandarin-speaking adults are more dependent on FM and fine spectral cues than French-speaking adults when discriminating lexical tones. In the same study, the influence of exposure to a tonal language was also evidenced when discriminating non-linguistic (click-train) stimuli showing similar F0 variations as the original lexical tones. This result is consistent with training effects observed for pitch perception both at the behavioral (e.g., Micheyl et al., 2006; Chandrasekaran et al., 2007; Fitzgerald and Wright, 2011; Burnham et al., 2014a,b) and neural levels (e.g., Wong et al., 2007; Kraus and Chandrasekaran, 2010). However, the influence of training and language exposure on general perceptual processes (i.e., non-linguistic stimuli) depends on the characteristics of stimuli and task (see Bent et al., 2006). Overall, these studies are consistent with the notion that auditory experience shapes the perception of AM and FM cues. In light of these adult studies, it is reasonable to hypothesize that the language-specific perceptual reorganization for lexical tones observed in infants is concomitant with a change in the perceptual weight assigned to speech modulation cues.

To date, the effect of early linguistic experience on the perception of modulation cues is still largely unexplored. Only few studies have recently investigated the perception of AM and FM speech cues in 6-month-old infants learning French or English (Bertoncini et al., 2011; Cabrera et al., 2013, 2015; Warner-Czyz et al., 2014). These studies showed that French-learning infants are able to discriminate phonetic contrasts (voiced versus voiceless, and labial versus dental stop consonants) on the sole basis of the slowest (<16 Hz) AM cues in a small number of broad frequency bands. In other words, French-learning infants do not require FM and fine spectral cues to discriminate a French voicing or place of articulation contrast. However, fine spectral cues are required to discriminate an English vowel contrast. These results are compatible with the idea that during the first year of life, infants progressively rely more on the modulation features that are relevant to the phonology of their native language (i.e., AM in the case of French). However, developmental data on infants acquiring other (and phonologically distinct) languages are still lacking.

The present study investigated to what extent early language experience influences the perception of speech modulation cues. This was achieved by testing the discrimination abilities of 6- and 10-month-old French- or Mandarin-learning infants using vocoded lexical tones. More generally, this study examined whether the perceptual reorganization observed for lexical tones during the first year of life is associated with a *reorganization in the weighting of the modulation cues* relevant to the perception of the native-language phonological properties.

As in the pioneer studies on infants’ lexical-tone perception (i.e., Mattock and Burnham, 2006; Mattock et al., 2008), syllables /ba/ containing either rising or low Thai tones were used in the present study. The Thai contour-level contrast (rising versus low) is known to be more difficult to discriminate than a contour–contour contrast (rising versus falling) by non-lexical-tone users (Abramson, 1978; Gandour and Harshman, 1978; Burnham and Francis, 1997) because of the relative acoustic similarity between rising and low tones whose F0 trajectories are highly similar until the mid-point of the contour. As in Mattock and Burnham (2006) and Mattock et al. (2008), the Thai tone contrasts are non-native (i.e., not familiar stimuli) for French and Mandarin infant groups^1^. Thus, using Thai tone contrasts might reduce the effect of stimulus familiarity on tone perception. Although, language-specific experience with a particular set of tones influences lexical tone processing (see Gandour et al., 2002; Burnham et al., 2014a), the processing of voice-pitch variations is also influenced by the presence of lexical tone in the surrounding language irrespective of whether the voice-pitch variations are native (i.e., language-specific) or non-native when comparing lexical-tone and non-lexical-tone listeners (see Mattock and Burnham, 2006; Burnham et al., 2014a; Cabrera et al., 2014). Lexical-tone discrimination was studied using the same stimuli as in Cabrera et al. (2014) study assessing French and Mandarin adult listeners’ use of spectro-temporal speech cues. Original lexical tones were left intact or vocoded in order to selectively degrade FM cues and fine spectral details. Ten- and 6-month-old-infants learning either French or Mandarin were tested in the intact or the vocoded-speech condition using a visual habituation paradigm (Werker et al., 1998).

If the native-language speech reorganization is concomitant with a change in the perceptual weight of AM and FM cues, only 10-month-old infants learning French and Mandarin should show different discrimination patterns. A first experiment was designed to verify the following hypotheses: (1) only the French 10-month-olds would not discriminate the lexical tones in the Intact condition (as in Mattock and Burnham, 2006; Mattock et al., 2008), and (2) French 10-month-olds would be less impaired by the reduction of the FM cues and fine spectral details conveying the voice-pitch variations than Mandarin-learning infants of the same age (see Xu et al., 2002; Cabrera et al., 2014 for similar results in adults) and (3) 6-month-old infants would show a similar pattern of discrimination irrespective of their language group, if their perception of speech modulation cues has not been shaped yet by their linguistic experience. A second experiment was designed to explore whether the early language experience of French and Mandarin 10-month-old infants extends its influence upon the perception of F0 modulations to non-linguistic click-train signals conveying F0 variations (pitch trajectory) similar to those of the original lexical tones (i.e., Xu et al., 2002; Cabrera et al., 2014). Following Mattock and Burnham (2006), both Mandarin- and French-learning 10-month-old infants were expected to be able to detect the difference between those pitch-contrasted patterns. However, a large number of studies showed that adults speaking a tonal language usually better perform than speakers of non-tonal language in pitch contour processing (e.g., Bent et al., 2006; Xu et al., 2006; Swaminathan et al., 2008). Thus, if linguistic experience at 10 months has already affected the weight of the FM and fine spectral cues conveying F0 information (or pitch trajectory), only Mandarin-learning infants should discriminate F0-modulation variations.

## Experiment 1

Experiment 1 tested French-learning and Mandarin-learning 6- and 10-month-old infants on the lexical tone contrast /ba/ rising versus /ba/ low in two conditions (Intact vs. Vocoded).

### Participants

French-learning infants were recruited from a database at the University of Paris Descartes (Paris), and Mandarin-learning infants were recruited at the National Taiwan University (Taipei). Data from 64 10-month-old infants were analyzed in this experiment: 32 French-learning infants, 16 in the Intact condition (mean age = 309 days, range = 300–328 days; nine girls) and 16 in the Vocoded condition (mean age = 313 days, range = 302–333 days; 10 girls) and 32 Mandarin-learning infants, 16 in the Intact condition (mean age = 318 days, range = 296–333 days; seven girls) and 16 in the Vocoded condition (mean age = 319 days, range = 300–340 days; nine girls). Data from 64 6-month-old infants were also analyzed in this experiment: 32 French-learning infants, 16 in the Intact condition (mean age = 196 days, range = 177–213 days; nine girls), 16 in the Vocoded condition (mean age = 201 days, range = 183–208 days; five girls), and 32 Mandarin-learning infants, 16 in the Intact condition (mean age = 194 days, range = 167–208 days; five girls) and 16 in the Vocoded one (mean age = 194 days, range = 171–209 days; eight girls).

All families were informed about the goals of the current study and provided written informed consent before their participation, in accordance with the current French and Taiwanese ethical requirements. All infants were born full-term, without any history of medical complications. All infants had normal hearing (based on parental report of newborn-hearing screening results). Fifty additional 10-month-old infants participated in the study, but were not included for the following reasons: fussing and crying (*n* = 45), and failure to conform to the a posteriori habituation criteria (*n* = 5). The a posteriori habituation criteria consisted of a minimum of 20 s and a maximum number of habituation trials (calculated as the group mean + 2 SD, the habituation time being susceptible to vary according to the sound condition, see Cabrera et al., 2015). Forty-nine additional infants participated in the study, but were not included for the following reasons: fussing and crying (*n* = 45) and failure to conform to the a posteriori habituation criteria (*n* = 4).

### Stimuli

A female native speaker of Thai produced several utterances of the syllable /ba/, with two different lexical tones: rising and low (i.e., rising F0 trajectory versus flat F0 trajectory; F0 range: 100–350 Hz for both tones). The speaker was asked to speak clearly in an adult-directed register (in order not to accentuate acoustic differences between lexical tones, Liu et al. (2007).

**FIGURE 1:**
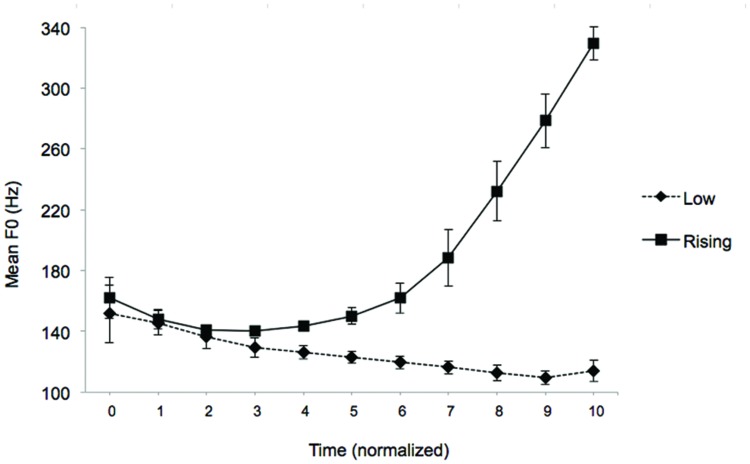
**Mean F0 (Hz) as a function of time.** F0 was averaged across the eight exemplars used in the low and the rising categories. Stimulus duration is normalized across exemplars.

In each category, eight different occurrences were chosen based on their clarity and duration. The mean duration of rising tones (661.6 ms, SD = 32.3 ms) and low tones (636 ms, SD = 31.2 ms) did not differ significantly [*t*(12) = 1.67, *p* = 0.13]. **Figure 1** represents the mean F0 variation calculated across the eight exemplars in each category. F0 variation is shown as a function of time, duration being normalized across exemplars (by intervals of 10% of the total duration). Two types of audio files were generated: a repeated sequence made of low tones only, and a repeated sequence made of rising tones only. Within each audio-file, tones were separated by a silent inter-stimulus-interval (ISI) varying randomly from 600 to 1300 ms. This variation was introduced to make small differences in duration between items irrelevant within and between categories. The total duration of each audio-file was around 26 s. Each file was constructed by taking four acoustically different tokens of a stimulus category repeated four times, for a total of 16 randomly ordered stimuli for the habituation sequences and the four others tokens were used for the test sequences. Four different random orders were created for both /ba/ rising and /ba/ low stimuli; two were used in the habituation phase, and the other two in the test phase.

In each speech condition, the AM and FM cues of the original speech signal were extracted within 32 or 8 frequency bands (spanning the range between 80 and 8020 Hz). The original speech signal was passed through a bank of either 32 2nd-order gammatone filters, each 1 equivalent-rectangular-bandwidth (ERB_N_) wide^2^, or of eight second-order gammatone filters each 4-ERB_N_ wide (Patterson, 1987; Gnansia et al., 2009). For the 32-band vocoder, the bandwidth and shape of analysis filters matched psychophysical estimates of auditory filter bandwidth and shape in normal-hearing listeners, and thus spectral cues were kept intact. In contrast, spectral cues were strongly degraded for the 8-band vocoder.

Then, in each band, AM and FM components were extracted using the Hilbert transform. The AM component was low-pass filtered using a zero-phase Butterworth filter (36 dB/octave rolloff) with a cutoff frequency set to ERB/2 in order to preserve the fast, F0-related AM fluctuations^3^. At this stage, the original FM carriers were either preserved (in the Intact condition) or replaced by sine-wave carriers with frequencies at the center frequency of the gammatone filters and with random starting phase in each analysis frequency band (in the Vocoded condition).

**FIGURE 2:**
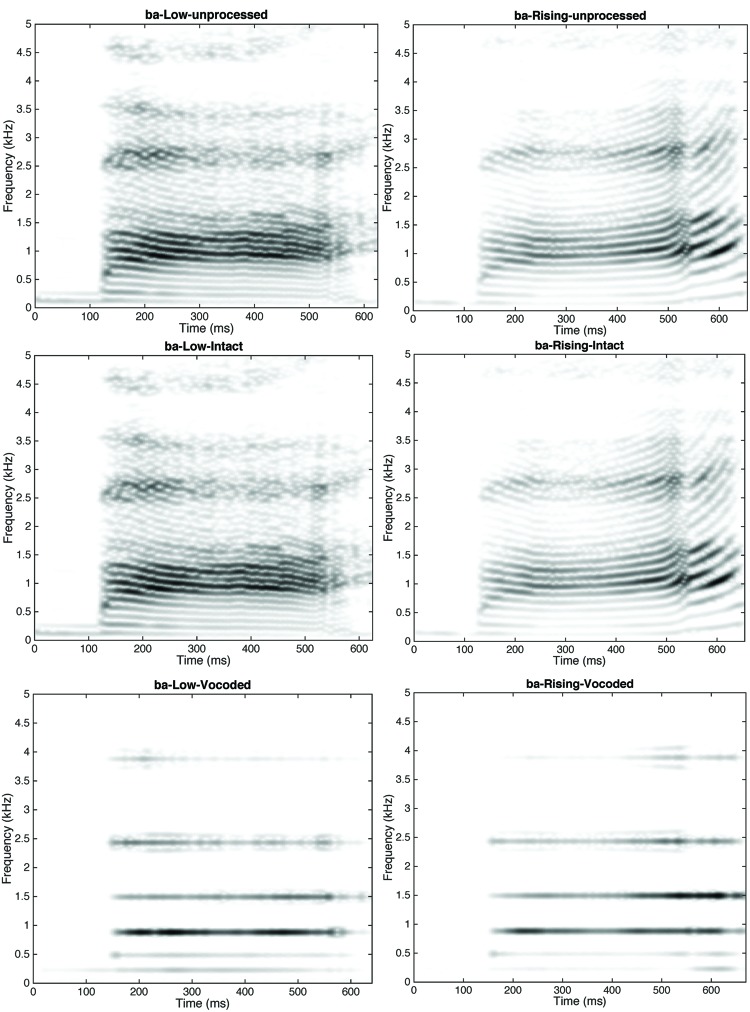
**Spectrograms of /ba/ rising **(left)** and /ba/ low **(right)** stimuli in the Original **(top)**, Intact **(middle)** and Vocoded **(bottom)** conditions**.

Finally, the AM and the carrier (either the original or sine-wave carriers) were recombined in each frequency band and the 32 or 8 modulated signals were summed. The level of the resulting speech signal was adjusted to have the same root-mean-square (rms) value as the input signal. **Figure 2** shows the spectrograms of the “/ba/low” and “/ba/rising” stimuli in each condition.

**FIGURE 3:**
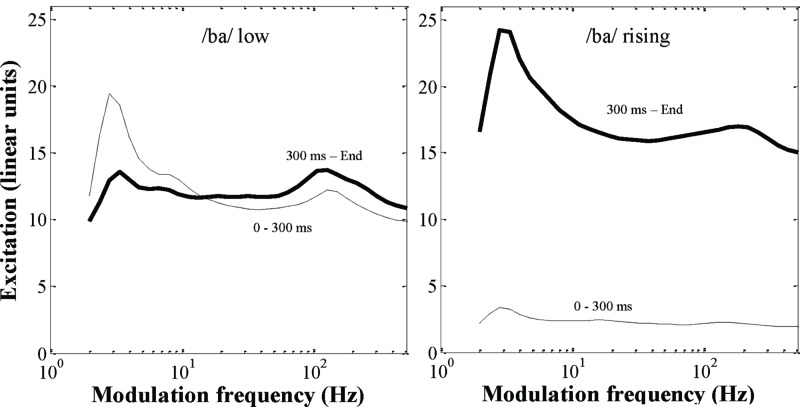
**Modulation excitation patterns computed for /ba/ low **(left)** and /ba/ rising **(right)** stimuli in the Vocoded condition.** Modulation excitation patterns show modulation power (in linear units) as a function of the center modulation frequency of modulation filters (ranging between 2 and 512 Hz). Modulation excitation patterns were computed for the highest (i.e., the eighth) analysis channel only (characteristic frequency = 6113 Hz). For each stimulus, modulation excitation patterns were computed for the first (thin lines) and last (thick lines) 300-ms time period of the stimulus to highlight (dynamic) changes in AM cues over time. The modulation excitation patterns shown here were obtained by averaging the modulation excitation patterns of the eight utterances for each stimulus category.

Thus, in the Intact condition, signal processing resulted in near-perfect stimulus reconstruction. In the Vocoded condition, the FM cues and fine spectral details of speech signals were severely degraded. Still, detailed analyses of AM patterns and AM modulation spectra for 8-band AM Vocoded stimuli indicate that low and rising /ba/ signals convey distinct AM cues that infants may use for discrimination. All the Vocoded stimuli (i.e., eight utterances for each stimulus category) were passed through a model of human AM perception corresponding to a simplified version of the “Envelope Power Spectrum Model” (EPSM model; Ewert and Dau, 2000). Simulations were conducted for the highest analysis channel (center frequency = 6113 Hz) of the 8-band AM vocoder only, because temporal-envelope cues elicited by sounds are better resolved by the more basal (and thus, broader) cochlear filters (see Appendix for a description of this model). For each stimulus category (/ba/ low versus /ba/ rising), a mean excitation pattern in the AM domain was computed over the eight utterances used in the present experiment, for the first and final 300 ms of the stimulus, respectively. The simulation results are presented in **Figure 3**. This figure shows that the Vocoded versions of /ba/ low and /ba/ rising stimuli differ in terms of slow (2–4 Hz) and fast (100–200 Hz) AM cues. As for slow AM cues, both /ba/ low and /ba/ rising Vocoded stimuli show a distinct peak in the modulation spectrum between about 2–4 Hz (more specifically, around 3.3–4 Hz for /ba/ low, and 2.8–3.3 Hz for /ba/ rising stimuli). Importantly, modulation excitation tends to remain constant or decrease over time for /ba/ low stimuli, whereas it increases drastically over time for /ba/ rising stimuli. This dynamic cue is visible in the spectrograms shown in **Figure 2** (bottom), and corresponds to the fact that overall level (and thus, loudness) increases at the end of /ba/ rising stimuli. As for faster AM cues, both /ba/ low and /ba/ rising Vocoded stimuli show another (though less salient) peak in the modulation spectrum between about 100 and 200 Hz (more specifically, around 100–128 Hz for /ba/ low, and 150–180 Hz for /ba/ rising stimuli). In this region of the AM spectrum, excitation increases slightly over time for /ba/ low stimuli, and more strongly for /ba/ rising stimuli. Moreover, modulation excitation shifts toward slightly lower AM filters during the last 300 ms of /ba/ low stimuli. In comparison, modulation excitation shifts toward higher AM filters during the last 300 ms of /ba/ rising stimuli. These dynamic changes in AM rate reflect the increase in voice-pitch at the end of the stimulus of /ba/ rising stimuli only (cf. **Figure 1**). Thus, although the AM vocoder severely reduces the FM cues and fine spectral details of the original lexical tones, it preserves loudness and pitch cues that infants may use for discrimination.

### Procedure

French-learning babies were tested in Paris (France), and Mandarin-learning babies were tested in Taipei (Taiwan). In each location, infants were seated on the caregiver’s lap, approximately 2 m from the TV monitor, in a sound-proof booth. A video camera positioned below the stimulus monitor was linked to another screen outside the booth that was used to observe the infant’s looking behavior online. Two loudspeakers located on each side of the infant’s monitor, approximately 30° to the left and right of the centerline of the caregiver’s chair, delivered the auditory stimuli at a level of 70 dB SPL. The observer was unaware of the audio file presented. She recorded the duration of the infant’s looking time (LT) by pressing a key and controlled stimulus presentation using Habit X.10 (Cohen et al., 2000). The caregiver was instructed not to interfere with the infant’s behavior (i.e., not to point to the screen at any time) and wore headphones delivering masking music.

The present study used a “visual habituation” method to assess discrimination in infants (e.g., Werker et al., 1998; Houston et al., 2007; Mattock et al., 2008; Narayan et al., 2010). Audio files were presented contingently upon the infants’ looking orientation at a display (a black and white checkerboard) on the TV monitor. Auditory and visual presentations continued until the infant looked away for 2 s (automatically calculated by the computer *via* the experimenter who released the key press as soon as infants looked away) or at the end of the audio file (maximum 26 s). At the end of the trial, the checkerboard disappeared and flashing balls appeared to draw the infant’s attention back to the TV monitor. No auditory stimulus was presented during this interval between trials. Once the infant looked at the screen, the experimenter initiated the next trial. The experiment began with a habituation phase, during which infants heard the same sound category (either /ba/ low or /ba/ rising). The habituation phase ended when the mean LT on three consecutive sequences decreased by 50% compared to the longest three consecutive trials from a sliding window (online habituation criterion). The test phase directly ensued, during which infants received four novel (N) and four familiar (F) trials presented alternatively with the order counterbalanced across subjects (such as N-F-N-F-N-F-N-F or F-N-F-N-F-N-F-N). In each condition, half of the infants were habituated to rising tones and the other half were habituated to low tones. The video recording of each infant was then coded off-line by the experimenter (the same for both French and Mandarin infants) who was unaware of which test trial was novel or familiar. Infants who did not conform to the a posteriori habituation criteria (i.e., minimum of 20 s and maximum of number of trials corresponding the group mean + 2 SD) were then excluded from the analyses.

### Results

**FIGURE 4:**
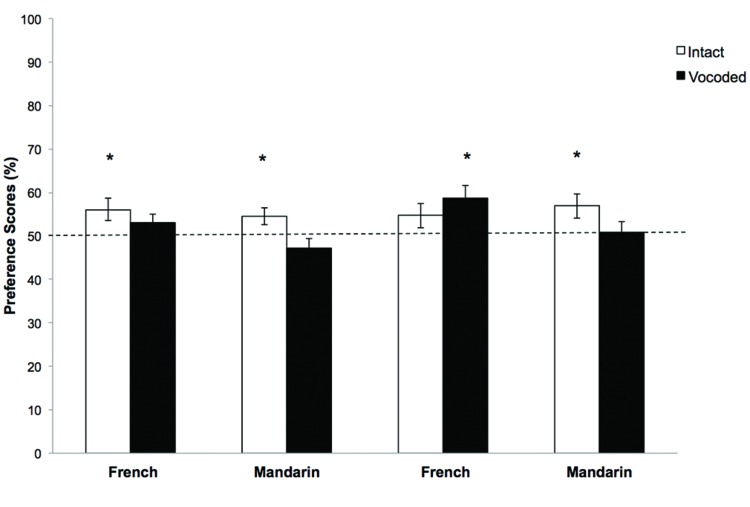
**Mean preference scores for novel trials in the test phase in the two signal-processing conditions (Intact, white bars and Vocoded, black bars) for 6-month-olds **(left)** and 10-month-olds **(right)**.** Data are shown for 10-month-old infants learning French and Mandarin. The error bars represent SE. The dotted horizontal line indicates chance level (50% detection). Stars indicate preference scores significantly different from chance (*p* < 0.05).

The mean LT in the test phase was measured for each group of infants. A preference score (or discrimination index) was computed for each infant (Gava et al., 2008; Beach and Kitamura, 2011). This preference score corresponded to the total LT for the four novel trials divided by the total LT for all eight-test trials (both familiar and novel trials). **Figure 4** represents the mean preference scores for each group. Two analyses were conducted in order to seek (1) the effect of language, age, and sound conditions on the preference scores, (2) to test whether the novel sequences were listened to longer than the familiar ones in each group.

An analysis of variance (ANOVA) conducted on the infants’ preference scores showed no main effect or interaction with the factor of habituation stimulus (rising versus low). Thus, the data were collapsed across this variable in the following analyses.

A comparison between groups was then run on the preference scores in a 2 (Conditions: Intact versus Vocoded) × 2 (Languages: French versus Mandarin) × 2 (Ages: 6 versus 10 months) ANOVA. The analysis showed a marginal effect of Condition [*F*(1,120) = 3.07, *p* = 0.08, η^2^ = 0.025], a marginal effect of Language [*F*(1,120) = 3.51, *p* = 0.06, η^2^= 0.028] and a significant interaction between Language and Condition [*F*(1,120) = 4.27, *p* = 0.04, η^2^= 0.034]. No other main effects or interactions were observed. Thus, the preference scores tend to vary across stimulus condition depending on native language. This indicates that Mandarin infants tend to be more affected than French by the reduction of the spectro-temporal fine structure speech cues.

Then, in order to determine whether infants were able to discriminate above chance (50% of total LT) novel sequences, one-sample *t*-tests were applied in each condition and for each language and age group. In the Intact condition, the Mandarin 6-month-olds showed a preference for novelty significantly above chance [mean = 54.6%, SD = 7.9; *t*(15) = 2.32, *p* = 0.035] as the French 6-month-olds [mean = 56.1%, SD = 10.4; *t*(15) = 2.32, *p* = 0.034]. The Mandarin 10-month-olds showed a preference for novelty significantly above chance [mean = 58.8%, SD = 11; *t*(15) = 2.49, *p* = 0.025], while the French 10-month-olds did not show a preference above chance for novel sounds [mean = 54.7%, SD = 11.2; *t*(15) = 1.67, *p* = 0.12].

In the Vocoded condition, a significant preference for novelty was only observed in the French 10-month-olds [mean = 58.8%, SD = 11.5; *t*(15) = 3.04, *p* = 0.008] but not in the Mandarin 10-month-old infants [mean = 50.8%, SD = 10.1, *t*(15) = 0.33, *p* = 0.75], French 6-month-olds [mean = 53.04%, SD = 7.6; *t*(15) = 1.6, *p* = 0.13], or Mandarin 6-month-olds [mean = 47.2%, SD = 8.5; *t*(15) = -1.31, *p* = 0.21]. These results are consistent with the previous overall analysis suggesting a different pattern of response between French and Mandarin according to the sound condition. However, this analysis reveals that the Language × Condition interaction is mainly driven by 10-month-old infants’ responses. Six-month-old babies of both languages tend to discriminate the Intact stimuli but not their Vocoded versions. Thus, they show similar patterns of preference to Mandarin 10-month-old infants.

### Discussion

Experiment 1 investigated the perception of lexical tones in 6- and 10-month-old infants learning a tone language or not. Overall, the linguistic experience of infants influences the responses to the Intact and Vocoded lexical tones. Mandarin infants were more affected by the reduction of the FM cues and fine spectral details when discriminating the Thai lexical tones than French infants. More precisely, in the Intact condition where speech modulation cues were close to those in the original signal, both Mandarin and French 6-month-old infants and the Mandarin 10-month-old infants exhibited a significant discrimination response (above chance) to the contrast between Thai low and rising lexical tones. This result is consistent with previous studies on lexical-tone perception in infants using difficult contrasts for non-tonal-language listeners (Mattock and Burnham, 2006; Mattock et al., 2008; Yeung et al., 2013). At 6 months, infants may not have *fully* reorganized their perception for lexical tones and 4 months later, unlike French-learning infants, Mandarin 10-month-old infants were able to discriminate a non-native Thai lexical tone presumably because of their experience with (linguistically relevant) pitch variations at the syllable level.

In the Vocoded condition, the FM cues and fine spectral details conveying salient information about F0 variations were severely degraded. In this condition, an opposite pattern of results was observed compared to the Intact condition. Only French 10-month-old infants discriminated the vocoded lexical tones whereas Mandarin 10-month-olds and both groups of 6-month-olds did not. Six-month-old infants required the spectro-temporal fine structure cues to discriminate lexical tones and could not use the AM cues only to discriminate voice-pitch patterns. At 10 months, only the performance of Mandarin-learning infants seems to be notably impaired with the degradation of FM cues and fine spectral details signaling F0 information, and thus, voice-pitch trajectories. Mandarin 10-month-old infants may have learnt to attend to and rely specifically on these spectro-temporal fine structure cues to process lexical-tone contrasts. Moreover, additional 4 months exposure to French may contribute to enhance the weight of AM speech cues or decrease the weight of FM speech cues, making 10-month-old infants able to discriminate vocoded lexical-tones. However, does the exposure to Mandarin enhance the weight of fine spectro-temporal cues conveying voice-pitch information of lexical tones? Mandarin-learning 10-month-old infants may have higher ability to use spectro-temporal fine structure cues to distinguish pitch trajectories than their French peers. A replication of the 10-month-olds’ results with stimuli containing only the FM cues related to the pitch trajectories in absence of linguistic information would provide evidence for linguistic experience influencing the auditory processing of FM and fine spectral cues.

A second experiment was designed to verify whether Mandarin 10-month-olds have better sensitivity to the FM cues related to F0 modulations found in lexical tones than their French peers. More precisely, discrimination of pitch trajectories conveyed by FM cues alone, in the absence of AM cues, in non-speech signals, i.e., click trains, was assessed in French and Mandarin 10-month-olds.

## Experiment 2

Experiment 2 aimed to evaluate the perception of the pitch trajectory or F0-modulation patterns in the absence of any other cues, and whether native language experience could influence the perception of F0-modulation cues at 10 months of age. A third condition was designed to extract the F0 trajectories of the rising and low lexical tones without any other speech-related cues. It was assumed that if exposure to a tonal language enhances the weight of fine spectro-temporal cues related to F0 modulations, as found in lexical tones, Mandarin-learning 10-month-old infants should be better in discriminating the contour differences embedded in click-train sounds compared to French-learning 10-month-olds.

### Participants

Two new groups of 10-month-old infants learning either French or Mandarin were tested. Data from 32 10-month-old infants were analyzed in this experiment: 16 French-learning infants (mean age = 316 days, range = 307–330 days; nine girls) and 16 Mandarin-learning infants (mean age = 311 days, range = 300–338 days; four girls). Thirty-nine additional infants participated in the study, but were not included for the following reasons: fussing and crying (*n* = 33), failure to reach the habituation criteria (same as in Experiment 1; *n* = 6). The high attrition rate observed in this condition might be related to the artificial (buzz-like) timbre of these stimuli.

### Stimuli and Procedure

**FIGURE 5:**
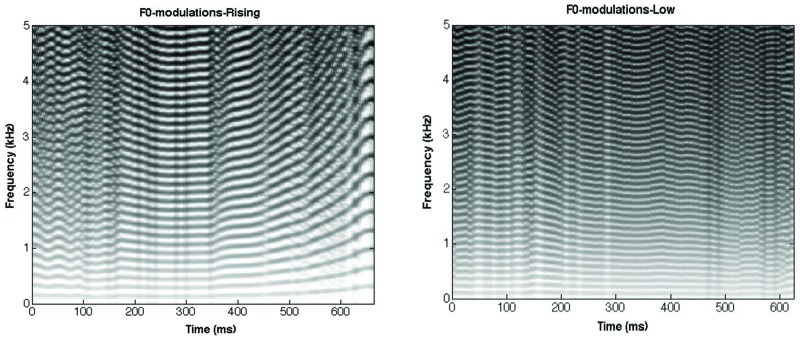
**Spectrograms of /ba/ low **(left)** and /ba/ rising **(right)** stimuli in the F0-modulation condition**.

The same original stimuli (eight exemplars of low and eight exemplars of rising Thai lexical tones) were used in the present experiment. The F0 trajectory of each original lexical tone was first extracted using the YIN algorithm (de Cheveigné and Kawahara, 2002). Then, this F0 trajectory was used to modulate the periodicity of a broadband click train (more precisely, the signal was a train of 88-microsecond-long square pulses, which were repeated at a rate equal to 1/F0). The click trains were limited to the frequency range between 80 and 22050 Hz, and were equated in rms power. **Figure 5** represents the spectrograms of these F0-modulation stimuli.

New audio files were generated with these stimuli with the same process as in Experiment 1. The procedure was the same as in Experiment 1 except that the test phase included four trials with two novel and two familiar trials because of the infants’ difficulty to maintain their attention to the stimuli (cf. high attrition rate).

### Results

**FIGURE 6:**
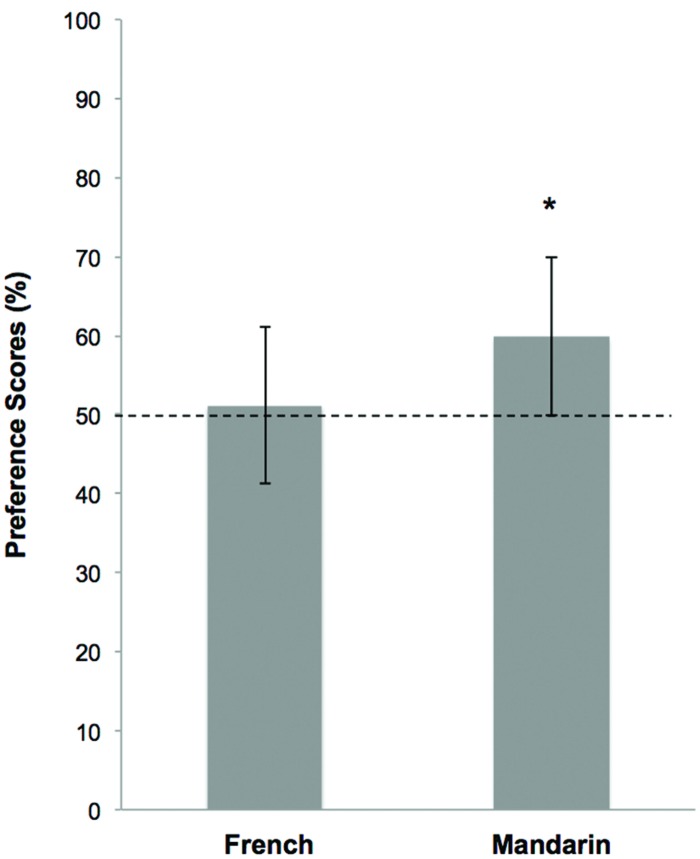
**Mean preference scores in the test phase in the F0-modulation condition**.

As in Experiment 1, preference scores were calculated for each language group (**Figure 6**). As in the previous experiment, there was no effect of the habituation stimulus (rising versus low) on preference scores. Thus, data were collapsed across this variable. A 2-way ANOVA (Languages: French versus Mandarin) revealed a marginal effect of Language [*F*(1,30) = 2.75; *p* = 0.108] on the preference scores.

Data are shown for the 10-month-old infants learning French and Mandarin. The error bars represent the SE. The dotted horizontal line shows chance level (50% detection). Stars indicate preference scores significantly different from chance (*p* < 0.05).

In order to assess whether the infants’ preference scores were significantly higher than chance level (50%), one-sample *t*-tests were calculated for each group to assess whether. The French 10-month-olds did not show a significant preference score for novel sequences [mean = 51.2%, SD = 14.4; *t*(15) = 0.33; *p* = 0.75]. However, the Mandarin-learning infants showed a significant preference for novelty [mean = 59.9%, SD = 15.4; *t*(15) = 2.58; *p* = 0.02]. In this F0-modulation condition, we observed the same pattern of results as that obtained in the Intact condition of Experiment 1. French- and Mandarin-learning 10-month-old infants presented with the same acoustic patterns did not show the same novelty preference, reflecting diverging processing of acoustic cues related to F0 contours.

### Discussion

The purpose of Experiment 2 was to compare the ability to discriminate two different categories of pitch trajectory (or F0-modulation patterns) in Mandarin-learning and French-learning 10-month-olds. FM cues related to F0 modulations were extracted from the original lexical tones and applied to click trains. In this F0-modulation condition, only Mandarin-learning infants were found to be able to discriminate the pitch trajectory. This finding differs from the results obtained by Mattock and Burnham (2006) who found that both English- and Mandarin-learning 10-month-old infants were able to discriminate F0 contours in musical tones played on a violin. In the present study, the F0 trajectories of the non-linguistic stimuli were derived from those of the linguistic stimuli and provided a more direct test of the ability to discriminate the F0 trajectories actually found in lexical tones, but in the absence of other linguistic information. Our results confirm that Mandarin-learning 10-month-olds are sensitive to the FM cues related to F0 trajectory of lexical tones unlike their French peers. These results together with those of Experiment 1 showing that Mandarin-learning 10-month-olds are not able to discriminate the vocoded lexical tones (that is, in the absence of salient voice-pitch cues conveyed by fine spectro-temporal cues) could be considered as the two sides of the same coin. At 10 months of age, Mandarin-learning infants demonstrate enhancement of their capacity to discriminate F0 trajectories conveyed by fine spectro-temporal cues compared to French-learning infants.

## General Discussion

The two experiments of the present study were designed to investigate whether basic sensory mechanisms (i.e., the auditory processing of spectro-temporal modulation cues) play a role in the perceptual reorganization of speech sounds observed during the first year of life.

In the present study, the discrimination of lexical tones (/ba/ rising vs. /ba/ low) similar to those used in recent developmental psycholinguistic investigations (Mattock and Burnham, 2006; Mattock et al., 2008) has been assessed using vocoded speech sounds altering selectivity spectro-temporal modulation cues for Mandarin- and French-learning infants.

### Early Changes in the Perceptual Weight of Spectro-Temporal Speech Modulation Cues

When the speech signals preserved the spectro-temporal modulation cues of the original lexical-tone signals (as in the Intact condition), Mandarin-learning infants discriminated the lexical-tone variations at both 6 and 10 months of age. However, in the Vocoded condition designed to reduce the spectro-temporal fine structure cues (FM and fine spectral details) conveying F0 cues, the 6- and 10-month-old Mandarin infants were not able to discriminate the vocoded lexical tones on the sole basis of temporal-envelope AM cues. These results suggest that for 6- and 10-month-old Mandarin-learning infants, spectro-temporal fine structure cues play a major role for discriminating pitch variations at the syllable level. It is possible that the perceptual reorganization for lexical tone has already started before 6 months (see Yeung et al., 2013). However, the present visual habituation procedure could not reveal whether infants learning Mandarin maintain their ability or become better at discriminating lexical tones in the Intact condition (and Thai lexical tones in particular) between 6 and 10 months of age. Indeed, they both discriminated the change in the test phase by looking longer to the novel sequences, but the procedure does not provide fine grained and comparable discrimination performance between groups (i.e., the response reflects only the presence or absence of discrimination). Thus, the present results do not indicate whether the spectro-temporal fine structure cues are more important at 10 months than at 6 months of age (Mandarin infants showing the same patterns of preference in both conditions).

In the Vocoded condition, the French 10-month-olds were found to be able to use the remaining AM speech cues. It may then be the case that Mandarin-learning 10-month-old infants are impeded by their linguistic experience to rely on this residual AM information. This pattern of results is reminiscent of those reported by Beach and Kitamura (2011) showing that English-learning 9-month-old infants are impaired in discriminating a native phonetic contrast compared to 6-month-olds who are unaffected by changes in the spectral profile of the speech stimuli.

Altogether, these results suggest that infants become more dependent on language-specific acoustic features over the 1st year of life. Moreover, the present cross-linguistic findings suggest that Mandarin infants specially attend to spectro-temporal fine structure cues that convey salient voice-pitch information.

The effect of linguistic experience on the perceptual weight of spectro-temporal fine structure cues was also explored in French-learning infants who have no prior experience with a tonal language but have 6 or 10 months of experience with the rhythm and prosody of French (i.e., syllable timed rhythm) that is mostly conveyed by AM cues (e.g., Rosen, 1992). Despite an overall improvement in auditory discrimination capacities (e.g., Saffran et al., 2006), French 10-month-old infants could no longer discriminate the pitch change that conveys the lexical meaning of syllables in the Intact condition, whereas French-learning 6-month-olds could. These results may reflect a developmental change in perceptual weighting of acoustic cues, that could correspond to reduced attention to voice-pitch variation within a syllable for French 10-month-olds (e.g., Conboy et al., 2008). These results replicate previous ones (e.g., Mattock and Burnham, 2006; Mattock et al., 2008), revealing a decline in the responsiveness to lexical tones after 6 months of age when pitch variations at the syllable level are irrelevant in the native language. On the other hand, in the Vocoded condition, 6-month-old infants were not able to rely only on AM cues to discriminate the lexical tones, while French-learning 10-month-old infants (as adults, see Cabrera et al., 2014) were more successful in using the remaining AM cues conveying rhythm-related information (i.e., duration, intensity). As suggested above for the Mandarin-learning infants, one possibility is that the longer experience with French prosody and phonological categories makes the 10-month-olds more likely to focus on the AM cues conveyed by the Vocoded stimuli and potentially relevant for French listeners (see Cabrera et al., 2013, 2015). In addition, one may speculate that French 10-month-old infants do not discriminate lexical tones in the Intact condition because they focus more on the AM cues than on the available spectro-temporal fine structure cues, although the latter convey more salient pitch cues. However, the present results also suggest that the reorganization of speech modulation cues may already start before 6 months of age. Indeed, the main analysis did not reveal an effect of age on the discrimination scores. This result may be related to the small number of participants tested. Younger infants (i.e., 4-month-olds) may show a stronger difference with 10-month-olds in their perception of lexical tones and use of speech modulation cues than 6-month-olds.

### Implications for Auditory Spectro-Temporal Processing

The present study also explored whether the reorganization of speech AM and FM processing corresponds to a general (non-linguistic) perceptual re-weighting of AM and FM cues.

The 10-month-olds’ results were replicated with non-linguistic (click-train) stimuli containing only the FM cues related to the voice-pitch trajectories of lexical tones. The discrimination results showed that only Mandarin-learning 10-month-old infants efficiently tracked F0 modulations conveyed by FM cues in both speech (i.e., Intact condition) and trains of clicks in the absence of any other speech-related information. In contrast with French-learning 10-month-olds, they seemed to specifically attend to FM cues to distinguish F0-trajectory information for both types of stimuli.

Overall, these findings suggest that the auditory perception of temporal modulation (AM and FM) cues underlying speech perception is flexible and affected by exposure to a specific auditory input (but see Elhilali et al., 2003; Jørgensen et al., 2013 for a hard-wired architecture perspective). This is consistent with training effects observed for pitch perception in musicians, lexical-tone users and trained adult subjects (e.g., Micheyl et al., 2006; Chandrasekaran et al., 2007; Fitzgerald and Wright, 2011; Burnham et al., 2014a,b). Moreover, cortical responses to spectro-temporal variations in acoustic stimuli have been shown to sharpen during development (e.g., Chang et al., 2005) and to be influenced by the auditory environment or task (e.g., Niwa et al., 2012; Bao et al., 2004, 2013). These psychoacoustic and neurophysiological studies highlight the plasticity of the auditory system for spectro-temporal modulation processing and the impact of the listening environment, in line with the present behavioral findings.

However, in the present study, the F0-trajectories applied on click-train signals were extracted from original speech signals. Thus, the present findings may result from the similarity of the non-linguistic and speech signals. In other words, it is still possible that the click-train signals may have been processed as (degraded forms of) linguistic signals. This is consistent with the results of Bent et al. (2006), that showed that non-tonal and tonal adult listeners behave similarly when linguistic and non-linguistic signals are totally unrelated. This is also consistent with the results of Mattock and Burnham (2006) showing no influence of language experience at 9 months of age when infants discriminate pitch variations produced by violin. Therefore, the extent to which language experience influences spectro-temporal processing should be explored using different kinds of non-linguistic sounds sharing more or less the acoustic complexity of original speech sounds.

## Conclusion

The present study shows that early linguistic experience with lexical tones does not only modulate the discrimination performance of speech contrasts, but that it also impacts the weight of spectral and temporal speech modulation cues during speech processing. These findings suggest that exposure to a tonal language may improve the listeners’ ability to track and use the pitch trajectory of lexical tone signaled by fine spectro-temporal modulation cues. Moreover, these findings also suggest that exposure to a syllable-timed language such as French improves the ability to use AM (temporal-envelope) speech cues.

Overall, this study provides an illustration of the early integrated and flexible relationship between different processing levels of auditory perception and reveals the interaction between the perceptual reorganization occurring around the first birthday for speech sounds and the auditory capacity to use spectro-temporal modulation cues.

## Conflict of Interest Statement

The authors declare that the research was conducted in the absence of any commercial or financial relationships that could be construed as a potential conflict of interest.

## Footnotes

1 Both Thai and Mandarin languages have complex tone systems with level and contour tones, but they differ in the number of tones (five and four, respectively), mean F0, F0 start, and F0 endpoint.

2 The ERB corresponds to the bandwidth of cochlear filters as measured for normal-hearing adults at moderate level (Glasberg and Moore, 1990).

3 Cochlear filtering (simulated here using gammatone filters) imposes limitations on the maximum AM rate. As in Gnansia et al. (2009), we adjusted the cutoff frequency of the lowpass filter (following gammatone filtering) to ERB_N_/2. This was achieved to ensure that the speech-envelope fluctuations transmitted by each gammatone filter were neither degraded (that is, too slow) nor perceptually irrelevant (that is, too fast). This was also intended to limit side-bands interaction between adjacent vocoder channels (and thus, beating effects).

## References

[B1] Abramson A. S. (1978). Static and dynamic acoustic cues in distinctive tones. *Lang. Speech* 21 319–325. 750791 10.1177/002383097802100406

[B2] Aslin R. N. Pisoni D. B. (1980). Some developmental processes in speech perception. *Child Phonol.* 2 67–96.

[B3] Aslin R. N. Pisoni D. B. Hennessy B. L. Perey A. J. (1981). Discrimination of voice onset time by human infants: new findings and implications for the effects of early experience. *Child Dev.* 52 1135–1145. 10.2307/1129499 7318516 PMC3499965

[B4] Bao S. Chang E. F. Teng C.-L. Heiser M. A. Merzenich M. M. (2013). Emergent categorical representation of natural, complex sounds resulting from the early post-natal sound environment. *Neuroscience* 248 30–42. 10.1016/j.neuroscience.2013.05.056 23747304 PMC3838508

[B5] Bao S. Chang E. F. Woods J. Merzenich M. M. (2004). Temporal plasticity in the primary auditory cortex induced by operant perceptual learning. *Nat. Neurosci.* 7 974–981. 10.1038/nn1293 15286790

[B6] Beach E. F. Kitamura C. (2011). Modified spectral tilt affects older, but not younger, infants’ native-language fricative discrimination. *J. Speech Lang. Hear. Res.* 54 658–667. 10.1044/1092-4388(2010/08-0177) 20844257

[B7] Bent T. Bradlow A. R. Wright B. A. (2006). The influence of linguistic experience on the cognitive processing of pitch in speech and nonspeech sounds. *J. Exp. Psychol. Hum. Percept. Perform.* 32 97–103. 10.1037/0096-1523.32.1.97 16478329

[B8] Bertoncini J. Nazzi T. Cabrera L. Lorenzi C. (2011). Six-month-old infants discriminate voicing on the basis of temporal envelope cues. *J. Acoust. Soc. Am.* 129 2761–2764. 10.1121/1.3571424 21568380

[B9] Best C. T. McRoberts G. W. LaFleur R. Silver-Isenstadt J. (1995). Divergent developmental patterns for infants’ perception of two nonnative consonant contrasts. *Infant Behav. Dev.* 18 339–350. 10.1016/0163-6383(95)90022-5

[B10] Burnham D. Francis E. (1997). “The role of linguistic experience in the perception of Thai tones,” in *Southeast Asian Linguistic Studies in Honour of Vichin Panupong* ed. Abramson A. S. (Bangkok: Chulalongkorn University press) 29–47.

[B11] Burnham D. Kasisopa B. Reid A. Luksaneeyanawin S. Lacerda F. Attina V. (2014a). Universality and language-specific experience in the perception of lexical tone and pitch. *Appl. Psycholinguist.* 77 571–591.

[B12] Burnham D. Brooker R. Reid A. (2014b). The effects of absolute pitch ability and musical training on lexical tone perception. *Psychol. Music* 1–17. 10.1177/0305735614546359

[B13] Cabrera L. Bertoncini J. Lorenzi C. (2013). Perception of Speech Modulation Cues by 6-Month-Old Infants. *J. Speech Lang. Hear. Res.* 56 1733–1744. 10.1044/1092-4388(2013/12-0169) 24023378

[B14] Cabrera L. Lorenzi C. Bertoncini J. (2015). Infants discriminate voicing and place of articulation with reduced spectral and temporal modulation cues. *J. Speech Lang. Hear. Res.* 58 1033–1042. 10.1044/2015_JSLHR-H-14-0121 25682333

[B15] Cabrera L. Tsao F.-M. Gnansia D. Bertoncini J. Lorenzi C. (2014). The role of spectro-temporal fine structure cues in lexical-tone discrimination for French and Mandarin listeners. *J. Acoust. Soc. Am.* 136 877–882. 10.1121/1.4887444 25096121

[B16] Chandrasekaran B. Krishnan A. Gandour J. T. (2007). Mismatch negativity to pitch contours is influenced by language experience. *Brain Res.* 1128 148–156. 10.1016/j.brainres.2006.10.064 17125749 PMC4372203

[B17] Chang E. F. Bao S. Imaizumi K. Schreiner C. E. Merzenich M. M. (2005). Development of spectral and temporal response selectivity in the auditory cortex. *Proc. Natl. Acad. Sci. U.S.A.* 102 16460–16465. 10.1073/pnas.0508239102 16263924 PMC1283465

[B18] Cheour M. Ceponiene R. Lehtokoski A. Luuk A. Allik J. Alho K. (1998). Development of language-specific phoneme representations in the infant brain. *Nat. Neurosci.* 1 351–353. 10.1038/1561 10196522

[B19] Cohen L. B. Atkinson D. J. Chaput H. H. (2000). *Habit 2000: A New Program for Testing Infant Perception and Cognition*. Austin: The University of Texas.

[B20] Conboy B. T. Sommerville J. A. Kuhl P. K. (2008). Cognitive control factors in speech perception at 11 months. *Dev. Psychol.* 44 1505–1512. 10.1037/a0012975 18793082 PMC2562344

[B21] de Cheveigné A. Kawahara H. (2002). YIN, a fundamental frequency estimator for speech and music. *J. Acoust. Soc. Am.* 111 1917–1930. 10.1121/1.1458024 12002874

[B22] Drullman R. (1995). Temporal envelope and fine structure cues for speech intelligibility. *J. Acoust. Soc. Am.* 97 585–592. 10.1121/1.413112 7860835

[B23] Elhilali M. Chi T. Shamma S. A. (2003). A spectro-temporal modulation index (STMI) for assessment of speech intelligibility. *Speech Commun.* 41 331–348. 10.1016/S0167-6393(02)00134-6

[B24] Ewert S. D. Dau T. (2000). Characterizing frequency selectivity for envelope fluctuations. *J. Acoust. Soc. Am.* 108 1181–1196. 10.1121/1.1288665 11008819

[B25] Fitzgerald M. B. Wright B. A. (2011). Perceptual learning and generalization resulting from training on an auditory amplitude-modulation detection task. *J. Acoust. Soc. Am.* 129 898–906. 10.1121/1.3531841 21361447 PMC3070992

[B26] Fu Q.-J. Zeng F.-G. Shannon R. V. Soli S. D. (1998). Importance of tonal envelope cues in Chinese speech recognition. *J. Acoust. Soc. Am.* 104 505–515. 10.1121/1.423251 9670541

[B27] Gandour J. T. Harshman R. A. (1978). Crosslanguage differences in tone perception: a multidimensional scaling investigation. *Lang. Speech* 21 1–33. 10.1177/002383097802100101 692240

[B28] Gandour J. Wong D. Lowe M. Dzemidzic M. Satthamnuwong N. Tong Y. (2002). A crosslinguistic fMRI study of spectral and temporal cues underlying phonological processing. *J. Cogn. Neurosci.* 14 1076–1087. 10.1162/089892902320474526 12419130

[B29] Gava L. Valenza E. Turati C. de Schonen S. (2008). Effect of partial occlusion on newborns’ face preference and recognition. *Dev. Sci.* 11 563–574. 10.1111/j.1467-7687.2008.00702.x 18576964

[B30] Glasberg B. R. Moore B. C. (1990). Derivation of auditory filter shapes from notched-noise data. *Hear. Res.* 47 103–138. 10.1016/0378-5955(90)90170-T 2228789

[B31] Gnansia D. Péan V. Meyer B. Lorenzi C. (2009). Effects of spectral smearing and temporal fine structure degradation on speech masking release. *J. Acoust. Soc. Am.* 125 4023–4033. 10.1121/1.3126344 19507983

[B32] Hoonhorst I. Colin C. Markessis E. Radeau M. Deltenre P. Serniclaes W. (2009). French native speakers in the making: from language-general to language-specific voicing boundaries. *J. Exp. Child Psychol.* 104 353–366. 10.1016/j.jecp.2009.07.005 19709671

[B33] Houston D. M. Horn D. L. Qi R. Ting J. Y. Gao S. (2007). Assessing speech discrimination in individual infants. *Infancy* 12 119–145. 10.1111/j.1532-7078.2007.tb00237.x 33412746

[B34] Jørgensen S. Ewert S. D. Dau T. (2013). A multi-resolution envelope-power based model for speech intelligibility. *J. Acoust. Soc. Am.* 134 436–446. 10.1121/1.4807563 23862819

[B35] Kong Y.-Y. Zeng F.-G. (2006). Temporal and spectral cues in Mandarin tone recognition. *J. Acoust. Soc. Am.* 120 2830–2840. 10.1121/1.2346009 17139741

[B36] Kraus N. Chandrasekaran B. (2010). Music training for the development of auditory skills. *Nat. Rev. Neurosci.* 11 599–605. 10.1038/nrn2882 20648064

[B37] Kuhl P. K. (2004). Early language acquisition: cracking the speech code. *Nat. Rev. Neurosci.* 5 831–843. 10.1038/nrn1533 15496861

[B38] Kuhl P. K. (2009). “Early language acquisition: neural substrates and theoretical models,” in *The Cognitive Neurosciences* ed. Gazzaniga M. S. (Cambridge, MA: MIT Press) 837–854.

[B39] Kuhl P. K. Stevens E. Hayashi A. Deguchi T. Kiritani S. Iverson P. (2006). Infants show a facilitation effect for native language phonetic perception between 6 and 12 months. *Dev. Sci.* 9 F13–F21. 10.1111/j.1467-7687.2006.00468.x 16472309

[B40] Kuhl P. K. Tsao F.-M. Liu H.-M. (2003). Foreign-language experience in infancy: effects of short-term exposure and social interaction on phonetic learning. *Proc. Natl. Acad. Sci. U.S.A.* 100 9096–9101. 10.1073/pnas.1532872100 12861072 PMC166444

[B41] Kuhl P. K. Williams K. A. Lacerda F. Stevens K. N. Lindblom B. (1992). Linguistic experience alters phonetic perception in infants by 6 months of age. *Science* 255 606–608. 10.1126/science.1736364 1736364

[B42] Liang Z. A. (1963). The auditory perception of Mandarin tones. *Acta Physiol. Sin.* 26 85–91.

[B43] Liu H.-M. Tsao F.-M. Kuhl P. K. (2007). Acoustic analysis of lexical tone in Mandarin infant-directed speech. *Dev. Psychol.* 43 912–917. 10.1037/0012-1649.43.4.912 17605524

[B44] Liu L. Kager R. (2014). Perception of tones by infants learning a non-tone language. *Cognition* 133 385–394. 10.1016/j.cognition.2014.06.004 25128796

[B45] Lorenzi C. Soares C. Vonner T. (2001). Second order temporal modulation transfer functions. *J. Acoust. Soc. Am.* 110 1030–1038. 10.1121/1.1383295 11519571

[B46] Mattock K. Burnham D. (2006). Chinese and English infants’ tone perception: evidence for perceptual reorganization. *Infancy* 10 241–265. 10.1207/s15327078in1003_3

[B47] Mattock K. Molnar M. Polka L. Burnham D. (2008). The developmental course of lexical tone perception in the first year of life. *Cognition* 106 1367–1381. 10.1016/j.cognition.2007.07.002 17707789

[B48] Micheyl C. Delhommeau K. Perrot X. Oxenham A. J. (2006). Influence of musical and psychoacoustical training on pitch discrimination. *Hear. Res.* 219 36–47. 10.1016/j.heares.2006.05.004 16839723

[B49] Narayan C. R. Werker J. F. Beddor P. S. (2010). The interaction between acoustic salience and language experience in developmental speech perception: evidence from nasal place discrimination. *Dev. Sci.* 13 407–420. 10.1111/j.1467-7687.2009.00898.x 20443962

[B50] Nittrouer S. (2002). Learning to perceive speech: how fricative perception changes, and how it stays the same. *J. Acoust. Soc. Am.* 112 711–719. 10.1121/1.1496082 12186050 PMC3987659

[B51] Niwa M. Johnson J. S. O’Connor K. N. Sutter M. L. (2012). Active engagement improves primary auditory cortical neurons’ ability to discriminate temporal modulation. *J. Neurosci.* 32 9323–9334. 10.1523/JNEUROSCI.5832-11.2012 22764239 PMC3410753

[B52] Patterson R. D. (1987). A pulse ribbon model of monaural phase perception. *J. Acoust. Soc. Am.* 82 1560–1586. 10.1121/1.395146 3693696

[B53] Pisoni D. B. (1977). Identification and discrimination of the relative onset time of two component tones: implications for voicing perception in stops. *J. Acoust. Soc. Am.* 61 1352–1361. 10.1121/1.381409 881488

[B54] PolkaL.ColantonioC.SundaraM. (2001). A cross-language comparison of/d/–/ 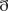 /perception: evidence for a new developmental pattern. *J. Acoust. Soc. Am.* 109 2190–2201. 10.1121/1.136268911386570

[B55] Polka L. Werker J. F. (1994). Developmental changes in perception of nonnative vowel contrasts. *J. Exp. Psychol. Hum. Percept. Perform.* 20 421–435. 8189202 10.1037//0096-1523.20.2.421

[B56] Ramus F. Nespor M. Mehler J. (1999). Correlates of linguistic rhythm in the speech signal. *Cognition* 73 265–292. 10.1016/S0010-0277(99)00058-X 10585517

[B57] Rivera-Gaxiola M. Silva-Pereyra J. Kuhl P. K. (2005). Brain potentials to native and non-native speech contrasts in 7-and 11-month-old American infants. *Dev. Sci.* 8 162–172. 10.1111/j.1467-7687.2005.00403.x 15720374

[B58] Rosen S. (1992). Temporal information in speech: acoustic, auditory and linguistic aspects. *Philos. Trans. R. Soc. Lond. B Biol. Sci.* 336 367–373. 10.1098/rstb.1992.0070 1354376

[B59] Saffran J. R. (2002). Constraints on statistical language learning. *J. Mem. Lang.* 47 172–196. 10.1006/jmla.2001.2839

[B60] Saffran J. R. Aslin R. N. Newport E. L. (1996). Statistical learning by 8-month-old infants. *Science* 274 1926–1928. 10.1126/science.274.5294.1926 8943209

[B61] Saffran J. R. Werker J. F. Werner L. A. (2006). “The infant’s auditory world: Hearing, speech, and the beginnings of language,” in *Handbook of Child Psychology* Vol. 6 eds Siegler R. Kuhn D. (New York, NY: Wiley) 58–108.

[B62] Seidl A. (2007). Infants’ use and weighting of prosodic cues in clause segmentation. *J. Mem. Lang.* 57 24–48. 10.1111/j.1467-7687.2008.00704.x

[B63] Seidl A. Cristià A. (2008). Developmental changes in the weighting of prosodic cues. *Dev. Sci.* 11 596–606. 10.1111/j.1467-7687.2008.00704.x 18576967

[B64] Shamma S. Lorenzi C. (2013). On the balance of envelope and temporal fine structure in the encoding of speech in the early auditory system. *J. Acoust. Soc. Am.* 133 2818–2833. 10.1121/1.4795783 23654388 PMC3663870

[B65] Shannon R. V. Zeng F. G. Kamath V. Wygonski J. Ekelid M. (1995). Speech recognition with primarily temporal cues. *Science* 270 303–304. 10.1126/science.270.5234.303 7569981

[B66] Sheft S. Ardoint M. Lorenzi C. (2008). Speech identification based on temporal fine structure cues. *J. Acoust. Soc. Am.* 124 562–575. 10.1121/1.2918540 18646999 PMC2809700

[B67] Smith Z. M. Delgutte B. Oxenham A. J. (2002). Chimaeric sounds reveal dichotomies in auditory perception. *Nature* 416 87–90. 10.1038/416087a 11882898 PMC2268248

[B68] Steeneken H. J. Houtgast T. (1980). A physical method for measuring speech-transmission quality. *J. Acoust. Soc. Am.* 67 318 10.1121/1.384464 7354199

[B69] Swaminathan J. Krishnan A. Gandour J. T. (2008). Pitch encoding in speech and nonspeech contexts in the human auditory brainstem. *Neuroreport* 19 1163–1167. 10.1097/WNR.0b013e3283088d31 18596621 PMC4373527

[B70] Tsao F.-M. Liu H.-M. Kuhl P. K. (2006). Perception of native and non-native affricate-fricative contrasts: cross-language tests on adults and infants. *J. Acoust. Soc. Am.* 120 2285–2294. 10.1121/1.2338290 17069324

[B71] Tsushima T. Takizawa O. Sasaki M. Shiraki S. Nishi K. Kohno M. (1994). “Discrimination of English/rl/and/wy/by Japanese infants at 6-12 months: language-specific developmental changes in speech perception abilities,” in *Proceedings of the Third International Conference on Spoken Language Processing* (Yokohama: DBLP).

[B72] Viemeister N. F. (1979). Temporal modulation transfer functions based upon modulation thresholds. *J. Acoust. Soc. Am.* 66 1364–1380. 10.1121/1.383531 500975

[B73] Warner-Czyz A. D. Houston D. M. Hynan L. S. (2014). Vowel discrimination by hearing infants as a function of number of spectral channels. *J. Acoust. Soc. Am.* 135 3017–3024. 10.1121/1.4870700 24815281 PMC4109213

[B74] Werker J. F. Shi R. Desjardins R. Pegg J. E. Polka L. Patterson M. (1998). “Three methods for testing infant speech perception,” in *Perceptual Development: Visual, Auditory, and Speech Perception in Infancy* ed. Slater A. (East Sussex: Psychological Press) 389–420.

[B75] Werker J. F. Tees R. C. (1984). Cross-language speech perception: evidence for perceptual reorganization during the first year of life. *Infant Behav. Dev.* 7 49–63. 10.1016/S0163-6383(84)80022-3

[B76] Werker J. F. Tees R. C. (2005). Speech perception as a window for understanding plasticity and commitment in language systems of the brain. *Dev. Psychobiol.* 46 233–251. 10.1002/dev.20060 15772961

[B77] Wong P. C. Skoe E. Russo N. M. Dees T. Kraus N. (2007). Musical experience shapes human brainstem encoding of linguistic pitch patterns. *Nat. Neurosci.* 10 420–422. 17351633 10.1038/nn1872 PMC4508274

[B78] Xu L. Pfingst B. E. (2008). Spectral and temporal cues for speech recognition: implications for auditory prostheses. *Hear. Res.* 242 132–140. 10.1016/j.heares.2007.12.010 18249077 PMC2610393

[B79] Xu L. Tsai Y. Pfingst B. E. (2002). Features of stimulation affecting tonal-speech perception: implications for cochlear prostheses. *J. Acoust. Soc. Am.* 112 247–258. 10.1121/1.1487843 12141350 PMC1414789

[B80] Xu Y. Gandour J. T. Francis A. L. (2006). Effects of language experience and stimulus complexity on the categorical perception of pitch direction. *J. Acoust. Soc. Am.* 120 1063–1074. 10.1121/1.2213572 16938992

[B81] Yeung H. H. Chen K. H. Werker J. F. (2013). When does native language input affect phonetic perception? The precocious case of lexical tone. *J. Mem. Lang.* 68 123–139. 10.1016/j.jml.2012.09.004

[B82] Yip M. (2002). *Tone.* Cambridge: Cambridge University Press.

[B83] Zeng F.-G. Nie K. Stickney G. S. Kong Y.-Y. Vongphoe M. Bhargave A. (2005). Speech recognition with amplitude and frequency modulations. *Proc. Natl. Acad. Sci. U.S.A.* 102 2293–2298. 10.1073/pnas.0406460102 15677723 PMC546014

