# Supplementary material for: The perception of speech modulation cues in lexical tones is guided by early language-specific experience
Source: Front Psychol. 2015 Aug 28;6:1290. doi: 10.3389/fpsyg.2015.01290 (PMC4551816; doi:10.3389/fpsyg.2015.01290)
Supplement: Supplementary file 1 [file Appendix.PDF]

APPENDIX

All the Vocoded stimuli used in experiments (*i.e.*, 8 utterances for each stimulus category) were passed through a model of human AM perception corresponding to a simplified version of the “Envelope Power Spectrum Model” (EPSM model; Ewert and Dau, 2000). Simulations were conducted for the highest analysis channel (center frequency=6113 Hz) of the 8-band AM vocoder only, because temporal-envelope cues elicited by sounds are better resolved by the more basal (and thus, broader) cochlear filters The input stimulus was initially passed through a 1-ERB_N wide bandpass predetection filter (6th-order butterworth filter) tuned to 6113 Hz. The filtered stimulus was half-wave rectified, and lowpass filtered (1st-order butterworth filter) at 150 Hz to simulate limited temporal resolution of the human auditory system (Viemeister, 1979). The resulting envelope was then passed through a bank of selective AM filters. This modulation filterbank was implemented as an array of overlapping linear bandpass filters whose center frequencies ranged between 2 and 512 Hz. Center frequencies were spaced on a logarithmic scale from 2 to 512 Hz, and filters’ density was set to 5 filters/oct. Filter bandwidths increased logarithmically over the whole range of center frequencies with constant Q value of 1 (Ewert and Dau, 2000; Lorenzi *et al.*, 2001). The modulation root-mean-square power was computed at the output of each modulation filter, resulting in a final “excitation pattern” in the AM domain.
